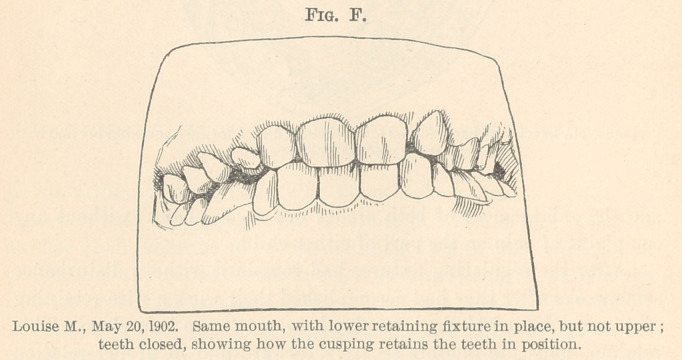# Observations on Some Recent Cases of Orthodontia

**Published:** 1902-12

**Authors:** E. A. Bogue

**Affiliations:** New York


### International Dental Journal.

Vol. XXIII. December, 1902. No. 12.

### Original Communications.[1]

### OBSERVATIONS ON SOME RECENT CASES OF ORTHODONTIA.[2]

BY E. A. BOGUE, M.D., NEW YORK.

The two cases that I have been observing are brother and sister, eight and nine years old respectively, and the observations have continued for a little more than two years. Both these children presented the curious anomaly of irregular deciduous teeth, though the teeth of both parents were, until extractions were practised, remarkably regular. This irregularity of the temporary teeth meant, of course, contracted arches. In the case of the boy the contraction was so great that the lips fell in like those of an edentulous person, giving him the appearance of a very old man. I greatly regret that I did not have a photographic profile made at the time, but his mother was so confident she had one that I did not urge the matter, and it is now too late to secure this almost unique specimen.

---

[1] The editor and publishers are not responsible for the views of authors of papers published in this department, nor for any claim to novelty, or otherwise, that may be made by them. No papers will be received for this department that have appeared in any other journal published in the country.

[2] Read before the Section on Stomatology, American Medical Association, Saratoga Springs, June 10, 1902.

The problem in both cases was to procure an enlargement of both arches in the easiest and most painless way possible, and with the least detriment to the teeth and disturbance to the children, and then to retain these teeth in their new positions, holding them out of one another's way during the continued shedding of the temporary teeth and the eruption of the permanent ones.

As children of that age are constantly active, fingers, tongue, and lips included, I determined upon fixed, that is to say, non-removable, apparatus, and the first was put on April 19, 1900, when the boy was just eight years old and the girl about nine.

Fig. 1.

The fixtures consisted of gold tubes soldered to rings, which were cemented on to the molar teeth, a wire screw bow carrying nuts at each end, the bows passing around in front of the incisors, to which they were attached by loops soldered to rings fitted over and cemented to the incisors, the ends of the bows being passed into the tubes on the molars. The position of the nuts on the ends of the bows was in front of the tubes. It will be perceived, therefore, that ligatures of any kind could be attached to any or all of the intervening teeth between the molars and the central incisors, which were the points of permanent attachment. The reason for putting two nuts on each end of these wire bows is that one nut may serve as a jam-nut to keep the other from turning backward after the pressure arising from its work has been relieved by the movement

FIG. 1 *A.*

John M., eight years old. Mouth as it was July, 1899, closed.

FIG. 1 *B.*

John M., eight years old. Mouth as it was July, 1899, open.

FIG. 1 *C.*

John M., July, 1899. Shows teeth closed and the upper molar far in advance of its proper position.

desired. In the case of the boy the upper incisors were so far within the arch as to make the child seem, as before stated, edentulous. Expansion was therefore begun on the upper jaw by turning the nuts on the wire bow a day or two after the rings had been cemented into place, the bow inserted into the tubes, and the patient had become accustomed to the apparatus being in place. About two weeks later a similar fixture was adapted to the lower teeth, this bow being attached to the two lateral incisors, this time by means of grass-line ligatures. Pressure was applied in both cases by turning the nuts, and in forty days such progress was made as rendered it quite safe for the boy to go away. He sailed for Europe June 6, 1900, with the regulating fixtures in place. One or two of the rings attached to the incisors came off and had to be recemented during his absence, but this was easily accomplished, and he returned from Europe and was presented for observation November 16, 1900. He had been absent five months and ten days, with very slight attention to the fixtures during that time.

In December, 1900, a gold wire retaining fixture was inserted to keep the lower incisors forward, where they had been drawn, on the principle that if the lower incisors were retained in position the upper ones could not fall back. Nothing was put upon the upper teeth. This wire retainer was broken and repaired from time to time, but was kept in place until January, 1902, thirteen months. Within a month from this date, as the bicuspids had begun to appear, rings and bars similar to those put on twenty months before were arranged to complete the expansion of the two arches above and below, and on February 6 and 10, the bars having been placed, the teeth were tied to them and traction was begun. On February 13 a gold screw was placed in the grinding end of the second left upper bicuspid, which tooth was partly erupted, three-quarters of its width inside the arch, as was also the right second bicuspid. This screw was wired to the bar. On March 10, one month from the time the fixtures were reapplied, it was discovered that the upper left permanent molar did not occlude properly with the lower, but that owing to an early loss of the second temporary molar it had come forward the full width of one cusp. As this occlusion would inevitably produce irregularity in the occlusion of all the teeth forward of that point on that side of the jaw, an upper rubber plate was inserted on March 17, 1902, which carried a screw resting in a long socket with a nut (afterwards changed to two nuts) on the screw, a claw on the end of the screw which rested against the upper molar above the line of the ring that was cemented to that molar. This claw prevented the screw from turning in the socket, and the turning of the nuts on the screw exerted a pressure backward against this permanent molar, which pressure was sustained by the entire roof of the mouth, contact against the incisor teeth beneath their rings, and the hold that the plate got under the ring attached to the right upper molar.

Fig. 6.

John M., March, 1902. Shows progress made in pushing backward the first left upper molar in about forty days, and exhibits fairly well the fixtures employed when he fell ill with measles and was not seen for one month.

Now began the most difficult part of this regulating for the boy. The force of eruption forward was so great that to obtain the slight movement backward that should allow the cusps of the left upper and lower molars to come into their proper places it was necessary to exert an opposing force that perceptibly changed (at least temporarily) the shape of the roof of the mouth. This pressure moved the rubber plate forward so that the scalloped points that penetrated between the teeth on that side of the mouth were moved to

FIG. 7 *A.*

John M., May 16, 1902. After recovery from measles. Shows conditions twenty-six days later, with left superior molar pushed back about three-thirty-seconds of an inch and the adjoining bicuspids drawn nearly to their proper position.

FIG. 7 *B.*

John M., May 16, 1902. Shows side view of teeth with mouth closed.

FIG. 7 *C.*

John M., May 16, 1902. Shows lower retainer in place the day boy sailed for Europe the second time.

the centres of the teeth, and the four incisors were driven forward nearly one-eighth of an inch.

FIG. *A*.

Louise M., four and one-half years old. Temporary teeth all in place; mouth closed.

On March 28 the second upper bicuspids, whose movements were commenced February 13, were found to be in place,—that is, suffi-

FIG. *B*.

Louise M. Same mouth and same date. Mouth open.

ciently drawn outward towards the bar not to require further movement. They were actually a little too far out. At this time measles attacked the children, and one month passed before I saw the patients again. During the eleven days that I had been trying to move the left upper molar backward, using only one nut, little progress was made, but on April 28 and 30 I resumed work by placing the two nuts in position and renewed operations upon the molar. In fourteen days the conditions were such as are evidenced by the models. The upper and lower left molars cusped with considerable accuracy, and I made my preparations to let the boy carry this regulating plate in his mouth again across the ocean. Previous

Fig. C.

Louise M., July, 1897. Same mouth, showing the first permanent molars and the incisors partly erupted.

to his sailing I constructed two upper rubber suction plates, designed to hold the upper molar where it is, to guide the bicuspids as they shall continue to erupt, inclining them a little outward of the lines of the normal arch, and for the time being to prevent the incisors from falling back, although they are now too far out. I have made a lower retaining apparatus that will scarcely more than touch the lower teeth at the gum margin, but that snaps in after the daily cleaning, and that will absolutely prevent the dropping back into a smaller arch of any of the teeth that have been drawn forward or outward. As soon, therefore, as the bicuspids shall have developed sufficiently for their long cusps to interlock the upper with the lower, the upper retaining plates may be discarded, for this cusping will surely hold the upper teeth in position so long as the lower teeth are firmly held. Having plenty of room, inasmuch as none of these teeth are in contact with each other, the development of the bicuspids was remarkably rapid. The models show how rapid.

The same plan was adopted for the little girl as for her brother,

Fig. D.

Louise M., December, 1900. Shows lower incisors drawn forward considerably and a beginning made on the upper incisors.

and the enlargement of both arches was accomplished without any complaint of pain on the part of either child.

After the regulating fixtures had remained without disturbance some weeks after they had accomplished their work a wire retaining plate was adapted to the lower teeth so as to snap into place. This retainer holds the incisors securely forward and in proper relation with the molars. It gives ample room for the development of the lower bicuspids and cuspids, and the upper teeth developing a little later will also have ample room to come to their respective places, because the upper incisors are held forward by shutting over the lower incisors, which are held firmly in position by the retainer.

FIG. E.

Louise M., May 20, 1902. Shows the mouth open, with the incisors both above and below advanced to their proper places and the lower retainer in position No retainer above.

FIG. F.

Louise M., May 20, 1902. Same mouth, with lower retaining fixture in place, but not upper; teeth closed, showing how the cusping retains the teeth in position.

I wish to emphasize the following facts:

First, that a perfectly arranged lower arch in ordinary cases guides the teeth of the upper arch into their proper positions and holds them there without additional appliances, and even in a case where the temporary teeth are irregular operations to correct deformity may be begun almost as soon as the first permanent molars and incisors make their appearance.

Second, that these operations may be almost or quite painless.

Third, that they may be accomplished with great rapidity and with such certainty that absence through unexpected sickness or protracted journeyings scarcely interrupts the orderly progress of the work.

Fourth, that this work may be accomplished with no perceptible detriment to the teeth.

Fifth, that the regulating fixtures themselves may be retained as retainers weeks or months after they have finished their corrective work.

Sixth, that retaining plates may be inserted that retain the lower teeth in position so surely that, as a rule, no retainer is needed above, if we have paid strict attention to the proper occlusion of the teeth.

Seventh, that these retaining fixtures may touch the teeth so slightly, as illustrated by Dr. Baker of Boston, that even if they are worn continuously without removal, the teeth can be as thoroughly cleansed around them as though the retaining fixtures were not in place.

## Figures and Tables

**Fig. 1. f1:**
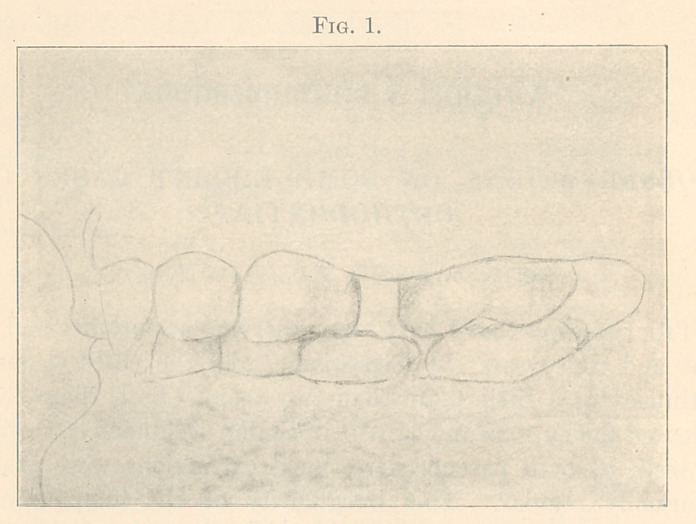


**Fig. 1 A. f2:**
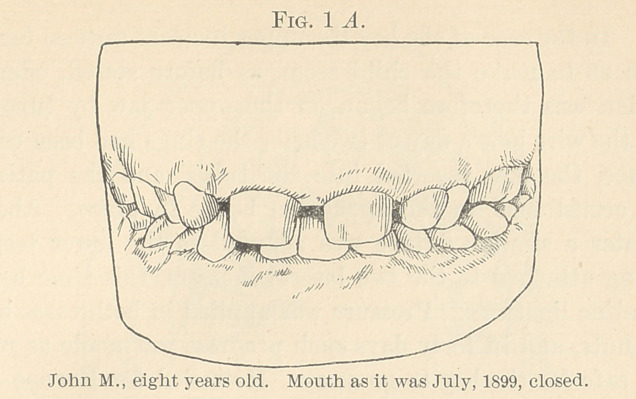


**Fig. 1 B. f3:**
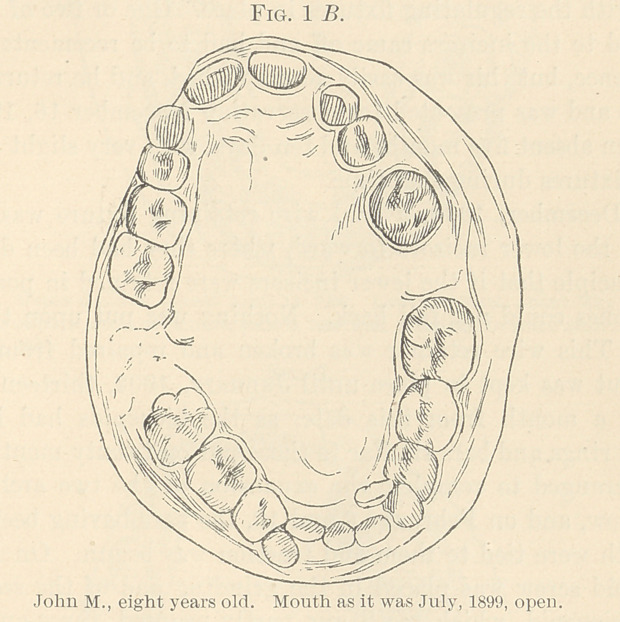


**Fig. 1 C. f4:**
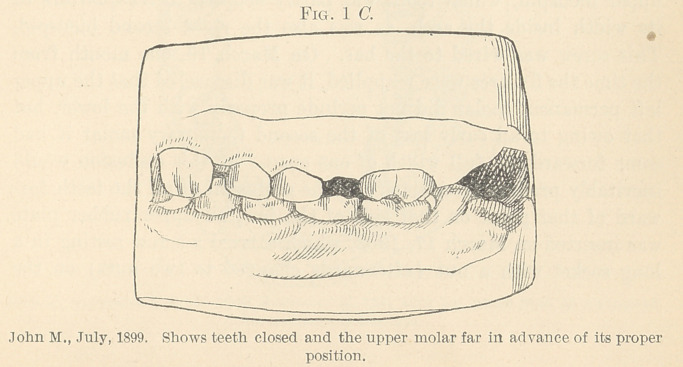


**Fig. 2. f5:**
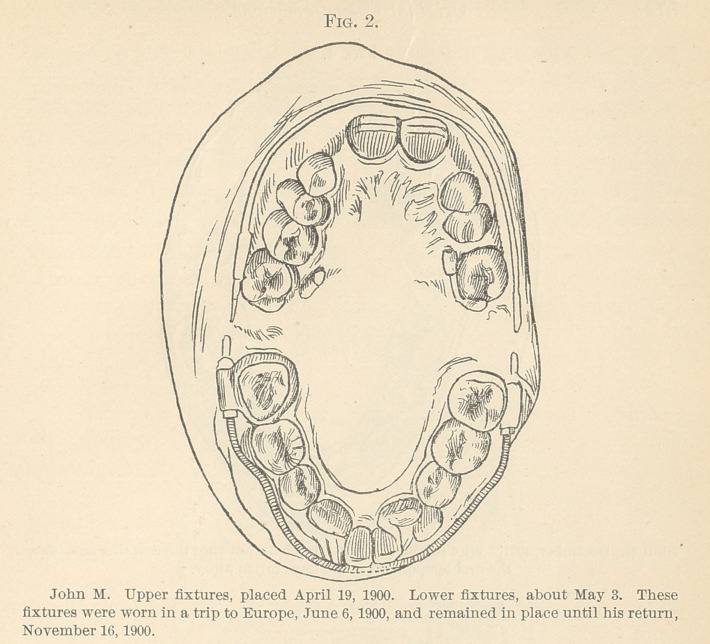


**Fig. 3. f6:**
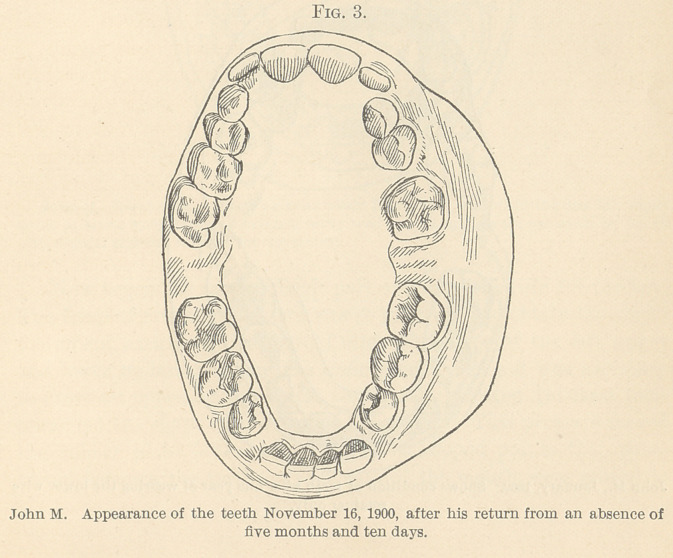


**Fig. 4. f7:**
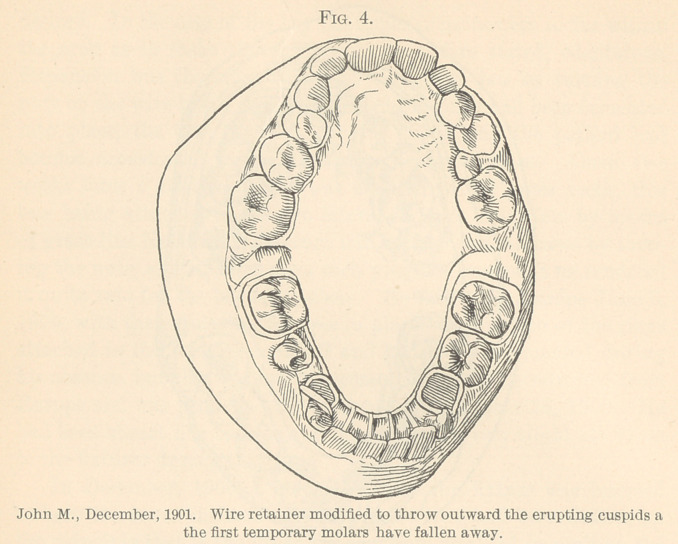


**Fig. 5. f8:**
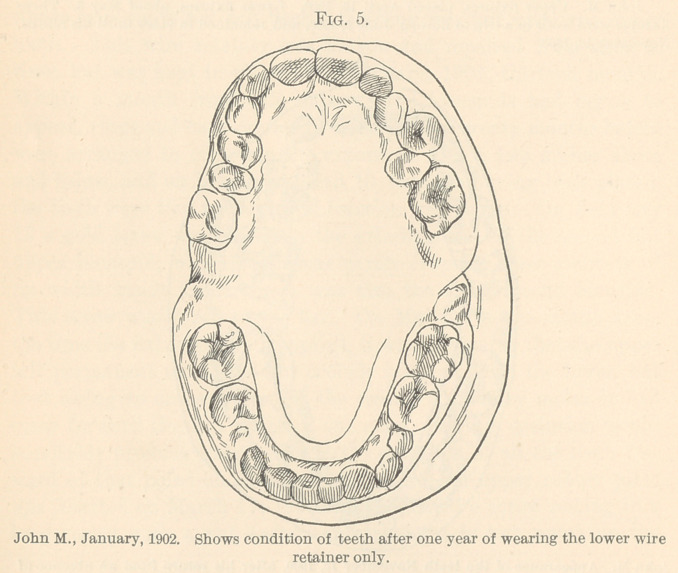


**Fig. 6. f9:**
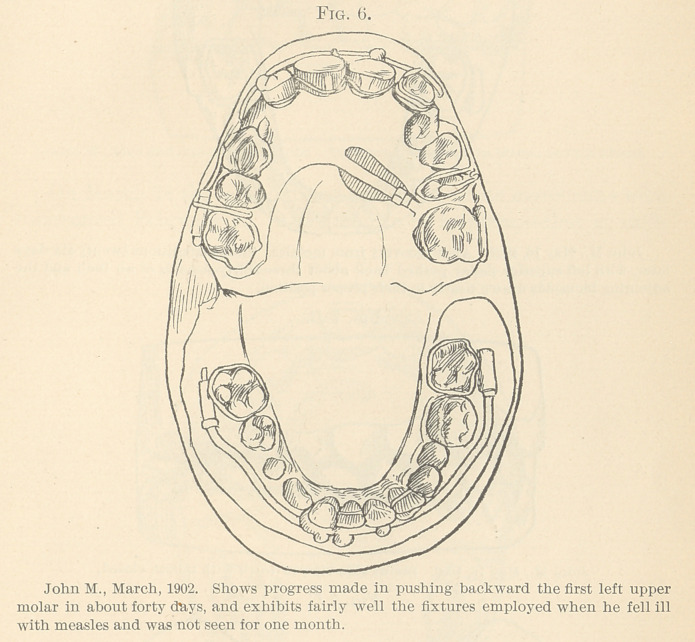


**Fig. 7 A. f10:**
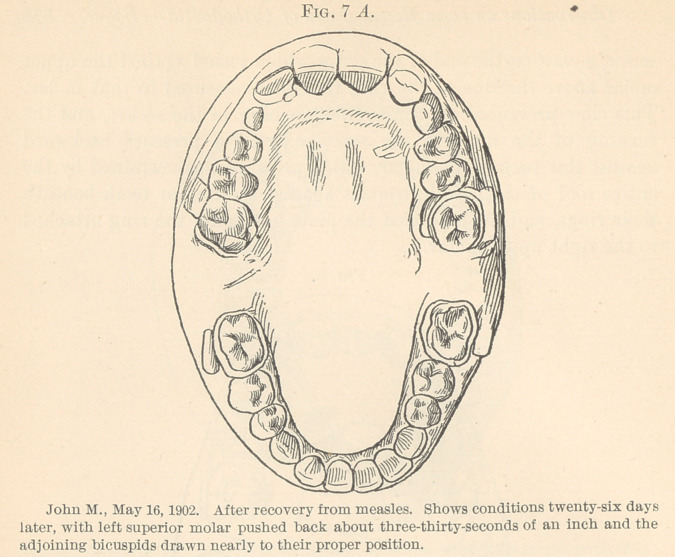


**Fig. 7 B. f11:**
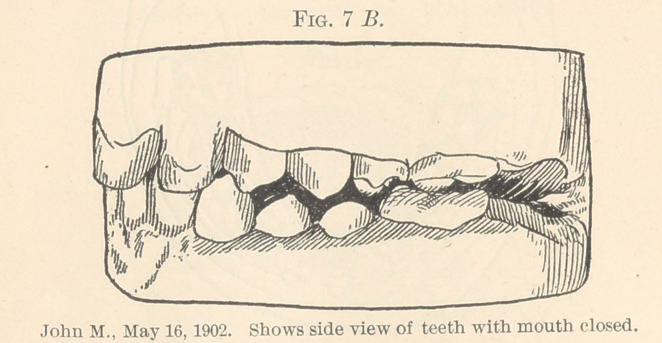


**Fig. 7 C. f12:**
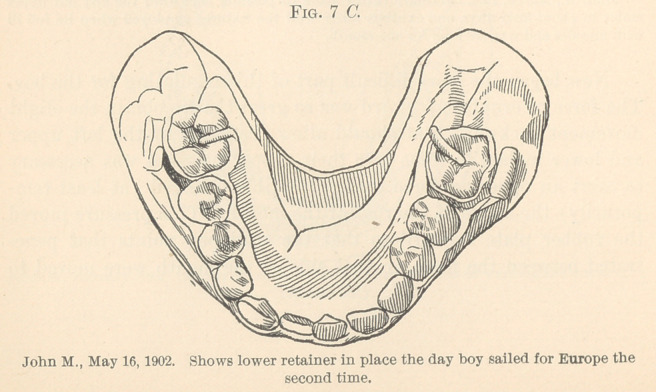


**Fig. A. f13:**
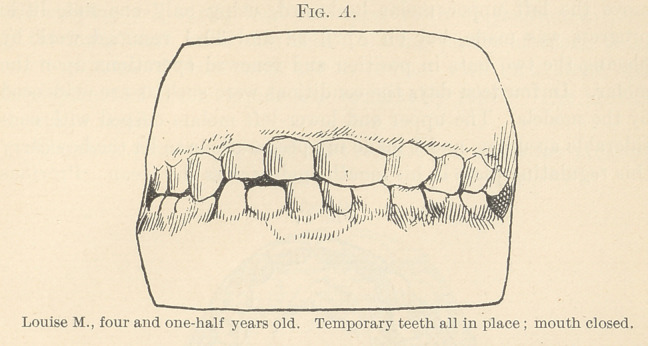


**Fig. B. f14:**
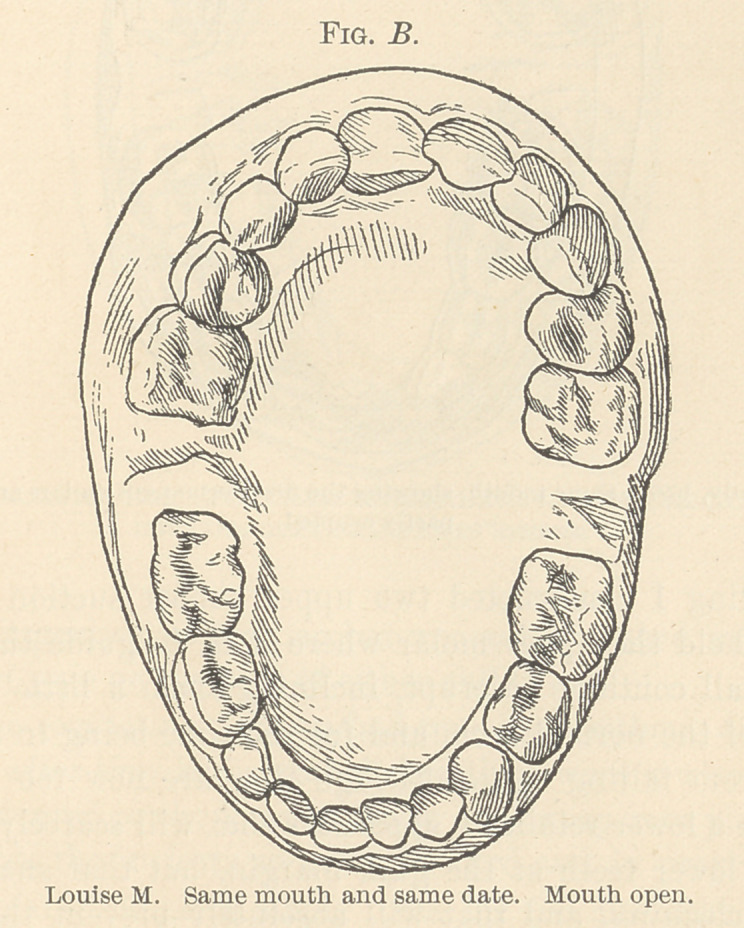


**Fig. C. f15:**
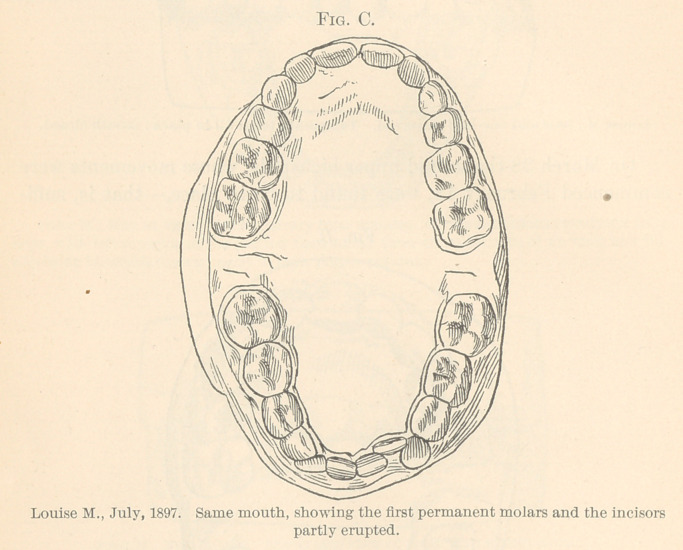


**Fig. D. f16:**
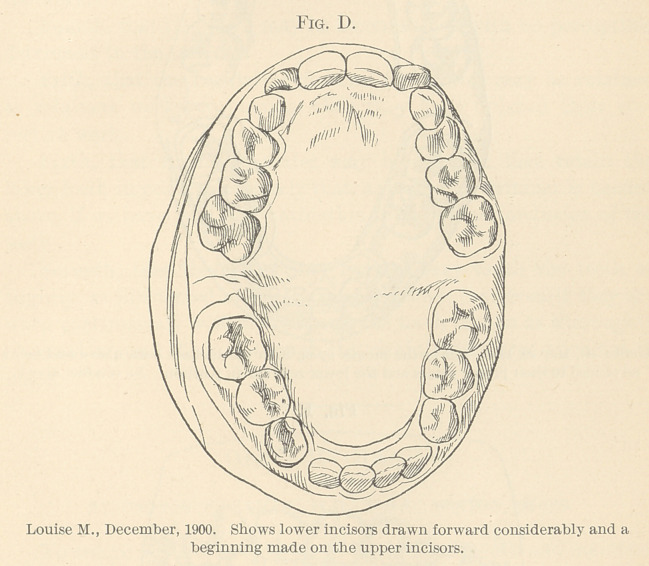


**Fig. E. f17:**
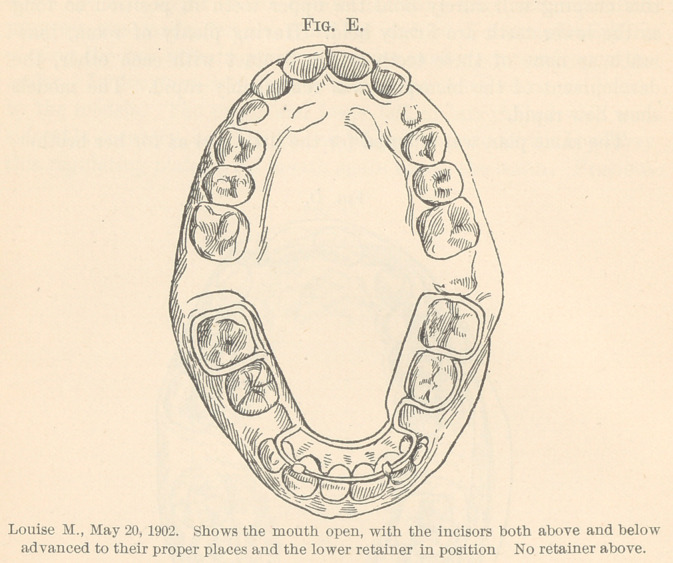


**Fig. F. f18:**